# Is sutureless thyroid surgery safe in the hands of surgical trainees. A single centre retrospective study

**DOI:** 10.1186/s13104-016-1940-7

**Published:** 2016-02-22

**Authors:** Peter C. Ambe, Dirk R. Wassenberg

**Affiliations:** Department of General, Visceral and Thoracic Surgery, St. Remigius Hospital Opladen, 51379 Leverkusen, Germany; Department of Surgery II, Helios Klinikum Wuppertal, Witten-Herdecke University, Heusnerstr. 40, 42283 Wuppertal, Germany

**Keywords:** Thyroid surgery, Thyroidectomy, Hypocalcaemia, Recurrent laryngeal nerve, Sutureless thyroid surgery

## Abstract

**Background:**

The safety and efficacy of sutureless thyroid surgery have been confirmed in many series. Equally, surgical expertise has been shown to influence postoperative outcome. This study aimed at investigating the safety of sutureless thyroid surgery in the hands of surgical trainees and to find out if this technique could be safely integrated into endocrine surgical training programs.

**Methods:**

A single center retrospective comparison of the outcome of surgeries performed by experienced surgical attendings and trainees was performed. The LigaSure Precise was used in all cases.

**Results:**

Two hundred and eight patients were included. The trainee group comprised of 61 cases managed by surgical trainees. Surgery was performed by surgical attendings in 147 cases (consultant group). The incidences of transient and permanent hypocalcaemia were 20.7 and 0.9 % respectively, the corresponding values for recurrent nerve injury were 6.3 and 0.9 %. Postoperative bleeding occurred in 3.4 %. There was no difference amongst both groups with regard to postoperative outcome (p > 0.05).

**Conclusions:**

While sutureless thyroid surgery was safely performed by surgical trainees without relevant increase in perioperative complications in our department, further larger scale studies would be needed to confirm this would be the case more widely.

## Background

With blood supply from four to five vessels, the thyroid gland represents one of the most vascularized organs of the human body. Its close proximity to neighboring structures, especially the recurrent laryngeal nerves (RLN) and the parathyroid glands, leaves limited room for surgical manipulation. Therefore hemostasis and injury to neighboring structures are of concern.

Over the last decade, sutureless thyroid surgery using sealing devices like the LigaSure^®^ Precise (Covidien, Boulder, CO, USA) has gained popularity [1–8]. This technique has been shown to be effective, safe and faster, with perioperative morbidities comparable to those of the “cut-and-tie” technique described by Kocher [9].

Since surgical expertise has been shown to influence postoperative outcome [10, 11], this study was design to investigate the influence of surgical expertise on postoperative morbidity in patients undergoing sutureless thyroid surgery with LigaSure^®^ and to verify if this technique could be safely integrated into endocrine surgical training programs.

## Methods

Following the approval of the hospital´s ethics committee at the St. Remigius Krankenhaus Opladen, Germany, the charts of patients undergoing thyroid surgery in the department of surgery from January 2011 until October 2013 were retrospectively reviewed. An informed consent was received from all patients for the use of their data in this study. Baseline characteristics and medical comorbidities as defined by the American Society of Anesthesiology (ASA) were retrieved for each patient. A written consent was obtained from each patient included in this study.

Perioperative data including the indication for surgery, surgical procedure, the duration of surgery, the course of surgery (visualization of the RLN, parathyroid glands and neuromonitoring signal) was retrieved from surgeon’s notes and surgical documentation sheets. All patients had a laryngoscopical examination before and after surgery. Postoperative calcium levels were retrieved from blood chemistry examination. Tissue weight, histopathology and data on the removal of parathyroid glands were retrieved from the final histopathology reports. All patients discharged with recurrent laryngeal lerve injury (RLNI) and hypocalcaemia were contacted for information about their present status with respect to the postoperative complications. The outcomes of patients operated upon by surgical trainees (trainee group) were compared to those of patients operated upon by experienced consultants (consultant group). Patients were consecutively assigned to a consultant surgeon or a surgical trainee based upon the availability of consultants/trainees. As part of our patient safety standards, cases with expected difficulties are primarily managed by experienced surgeons.

Statistical analysis was performed using the Statistical Package for Social Sciences, SPSS^®^, IBM, Version 21. The study population was described using absolute numbers, percentages, mean and standard deviations as needed. Significances were calculated using the Fisher’s exact test with the level of significance set at p < 0.05.

Primary endpoint was perioperative morbidity including: postoperative bleeding with the need of surgical intervention, hypocalcemia and RLNI. Secondary endpoints included: the duration of surgery and length of postoperative hospital stay.

Hypocalcemia was diagnosed in patients with typical clinical manifestations (paraesthesia, muscle spasm, Chovstek’s or Trousseau’s signs) and serum calcium levels below the normal institutional limits (2.10–2.43 mmol/l) needing either oral or intravenous (i.v.) calcium substitution. Postoperative recurrent laryngeal nerve injury was diagnosed following postopertive laryngoscopic examination, which was performed in all patients. Postoperative bleeding was defined as any form of bleeding in the field of surgery with the need of surgical intervention.

### Surgery

Surgery was performed either by an experienced consultant or by a surgical trainee (usually beginning in the 3rd year of training) under direct supervision. Following endotracheal intubation, the patient is placed in the supine position with the arms fixed by the sides. Using a head ring, the neck is sufficiently extended. A collar incision is made two fingers above the jungulum and carried through the platysma while hemostasis is done using a bipolar diathermy. Two stay sutures are placed on the superior subplatysmal flap on which saline bottles are attached to keep the situs open. The procedure then proceeds as described by O'Neill et al. [6].

The LigaSure^®^ Precise (Covidien, Boulder, CO, USA) was used in all cases. Besides direct visualization of the RLN, intraoperative neuromonitoring (IONM) was done in all cases before and after resection. Furthermore, IONM of the vagus was performed in all cases. Equally, the parathyroid glands were identified during surgery and left in situ. In patients undergoing thyroidectomy, resection of the contralateral lobe was postponed following abnormal IONM signal on the ipsilateral side. In such cases, surgery was completed following normal findings on laryngoscopy.

## Results

**Fig. 1:**
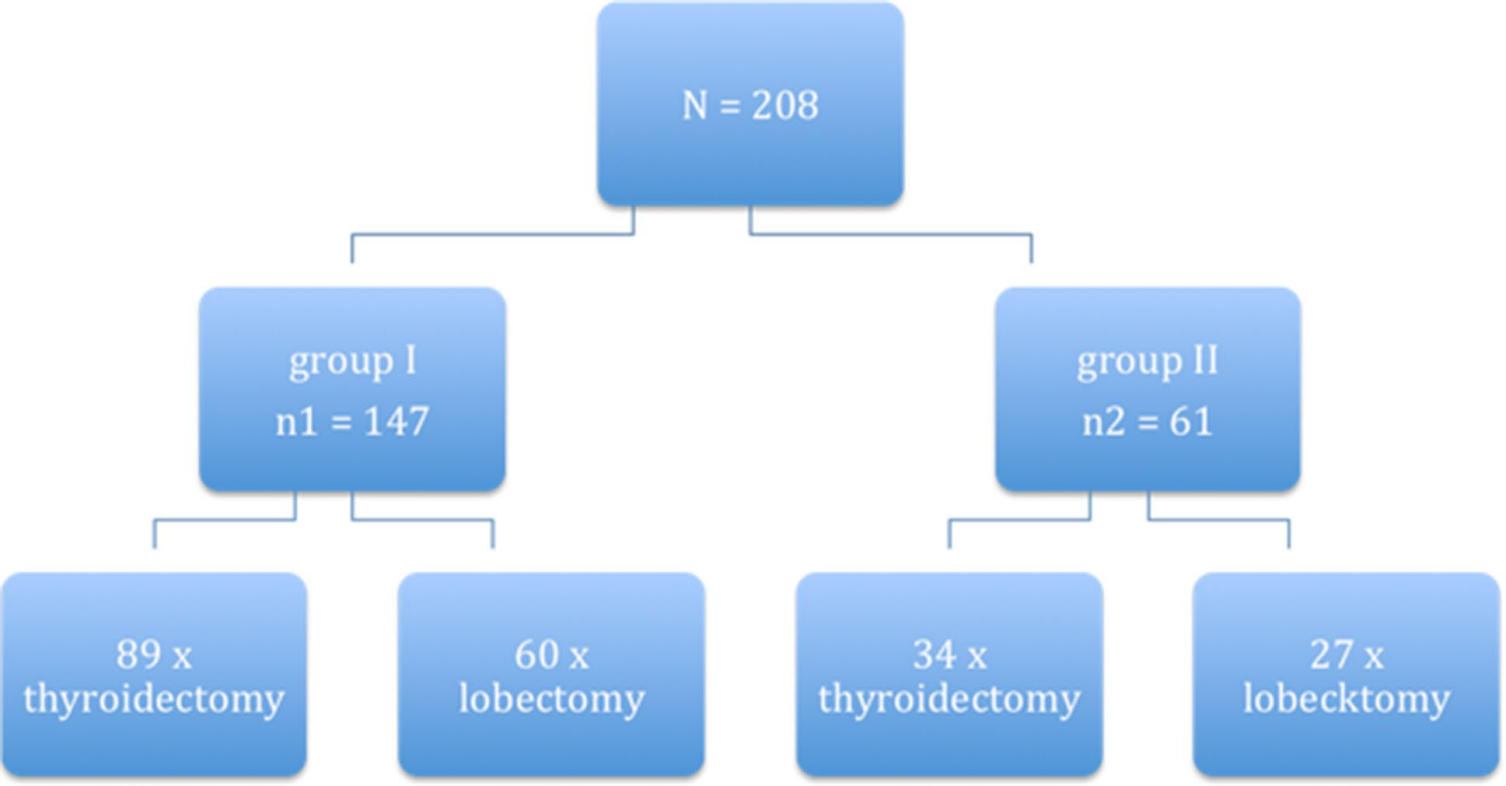
Distribution of the study population. The outcomes of patients managed by surgical trainees were compared to those of patients managed by consultants

Two hundred and eight patients underwent thyroid surgery within the period of investigation. The baseline characteristics of the study population are summarized in Table 1. Surgery was performed in 147 cases (70.7 %) by consultants (consultant group) and in 61 cases (29.3 %) by surgical trainees (trainee group), Fig. 1.

**Table 1:** Baseline characteristics of the study population

Characteristics	Consultant group	Trainee group	p value
Female/male	106/41	46/15	>0.05
Mean age (yrs)	58.1 ± 14.6	58 ± 11.7	>0.05
ASA			
1–2	117	51	>0.05
>2	30	10	
BMI (kg/m^2^)	27.8 ± 8,4	25 ± 11.4	>0.05

The demographic characteristics of the study population were similar in both groups

*Yrs* years, *BMI* body mass index

Nodular goiter was the most common indication for surgery. Thyroidectomy was performed in 58.2 % (121/208) of the study population while lobectomy was done in 41.8 % (87/208). There was no significant difference (p = 0.35) amongst both groups with respect to surgical diagnosis and procedure. However, a significant (p = 0.03) number of cases with large goiter were managed by consultants (47.6 ± 51.9 vs. 36.9 ± 21.3 g) Table 2.

**Table 2:** Summary of histopathology and surgical procedures

Features	Consultant group	Trainee group	p value
Histopathology			
Nodular goiter	97 (66.7 %)	37 (60.7 %)	>0.05
Adenoma	20 (13.6 %)	15 (24.6 %)	
Carcinoma	15 (10.2 %)	5 (8.2 %)	
Grave’s disease	8 (5.4 %)	2 (3.3 %)	
Hashimoto thyroiditis	6 (4.1 %)	2 (3.3 %)	
Surgical procedure			
Thyroidectomy	89 (59.2 %)	34 (55.7 %)	>0.05
Lobectomy	60 (40.8 %)	27 (44.3 %)	
Nerves at risk	238	95	/
Mean tissue weight	47.5 ± 51.9 g	36.8 ± 23.3 g	0.03

There was no significant difference amongst both groups with respect to histopathology and surgical procedure. The mean tissue weight was significantly higher in group I

**Fig. 2:**
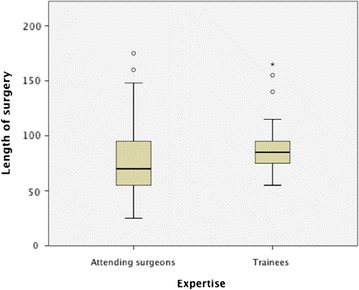
Duration of surgery. Surgical trainees operated significantly (p = 0.02) longer than attending surgeons

The mean duration of surgery in the general population was 78.0 ± 27.3 min. The mean duration of surgery was 74.8 ± 28.8 min in consultant group. This was significantly short (p = 0.02) compared to 85.9 ± 21.2 min in trainee group, Fig. 2. Intraoperative blood loss was comparable in both groups (60 ± 40 ml vs. 80 ± 23 ml, p = 0.27).

**Fig. 3:**
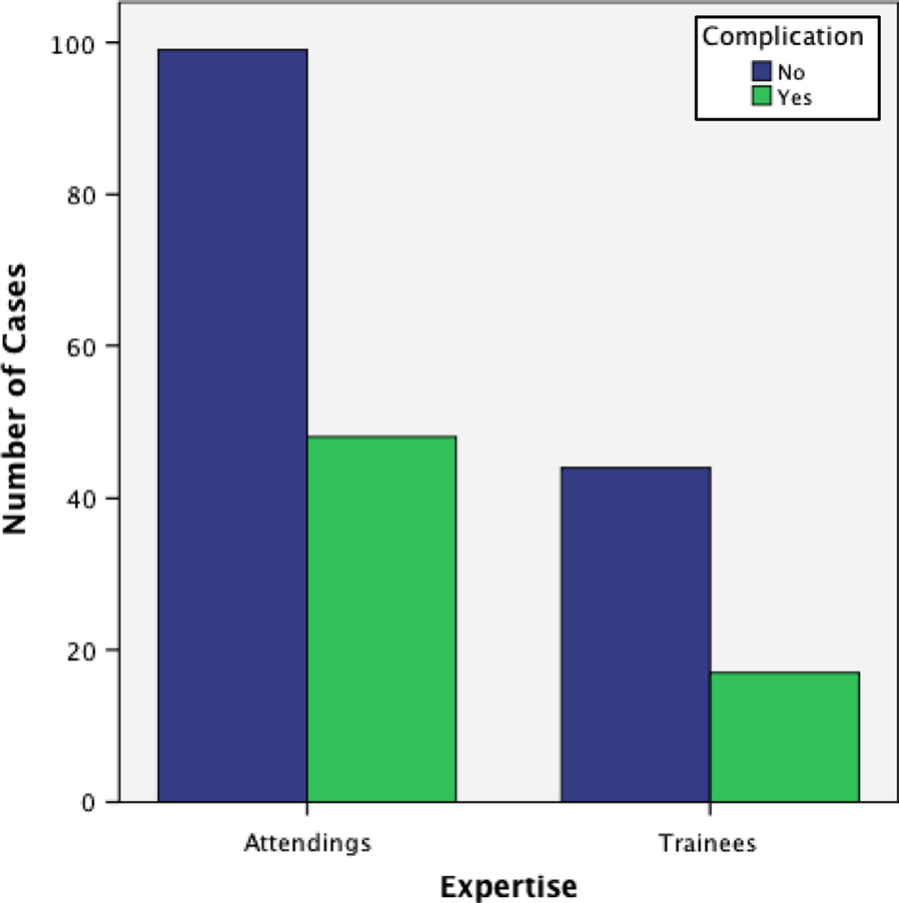
Postoperative complications. There was no significant difference (p = 0.4) in perioperative morbidity amongst both groups

The incidence of transient postoperative hypocalcemia in this series was 20.7 % (43/208). The corresponding incidences were 21.7 % (31/147) and 19.7 % (12/61) in consultant group and trainee group respectively. Permanent hypocalcemia developed in two cases (0.9 %), both from consultant group. The rate of postoperative hemorrhage was 3.4 % (7/208). Five of the seven cases of postoperative hematoma occurred in consultant group (3.4 %) while two cases occurred in trainee group (3.2 %). The incidence of transient RLNI, with respect to the number of lobes resected in this series was 6.3 %. The corresponding incidences were 6.7 and 5.2 % in consultant group and trainee group respectively. The incidence of permanent RLNI was 0.9 % (2 cases), both from consultant group. There was no significant difference (p = 0.41) amongst both groups with respect to postoperative complications, Fig. 3. The mean length of postoperative stay was 4 ± 1 day. This was similar in both groups. There was no mortality in this series.

## Discussion

Sutureless thyroid surgery has gained grounds in the last decade. The effectiveness and safety of suture devices like Harmonic-Scapel^®^ and LigaSure^®^ have been proven in many studies [1, 3, 5, 12–16]. Moreso, sutureless thyroid surgery has been shown to reduce operative time and overall cost of surgery [17]. Being a relatively new technique, little is known about the safety of this technique in the hands of surgical trainees.

The aim of this study therefore was to investigate the safety of sutureless thyroid surgery in the hands of surgical trainees. Using data from a single center, a retrospective analysis of the outcomes of patients managed by surgical trainees was compared to those of patients managed by consultants. In all cases, sutureless thyroid surgery was performed using the vascular sealing device LigaSure^®^ Precise, Covidien, Boulder, CO, USA. Both groups were comparable with regard to comorbidity, diagnosis and surgical procedures.

In our surgical training program, trainees start to perform thyroid procedures in the fourth year of their training. By this time, all our trainees must have assisted in quite a number of thyroid procedures. The consulting surgeons on the other hand had enough experience in thyroid surgery with >300 thyroid procedures each.

Trainees performed about 30 % of surgeries in this study. The mean duration of surgery in this series was 78.0 ± 27.3 min. This was comparable to published data for sutureless thyroid procedures [18–20]. As expected, surgery lasted significantly longer (p = 0.02) when performed by trainees.

The incidence of transient postoperative hypocalcemia requiring calcium and vitamin D substitution was 19.7 % in the trainee group and 21.7 % in the consultant group. The rate of permanent hypocalcemia in this series was 0.9 % (2 cases), both from the consultant group. The incidence of transient RLNI with respect to nerves at risk was 5.2 % in the trainee group and 6.7 % in the consultant group. Permanent RLNI was seen 0.9 % (2 cases), both from the consultant group. Similar rates have been reported in a recently published meta-analysis by Lang et al. [21–23].

Interestingly, IONM signals were normal in almost all cases with transient postoperative RLNI. Since the RLN was visualized and free of tension in the course surgery, recurrent laryngeal nerve dysmotility must be blamed on postoperative hematoma, even though drains were placed in all cases. This explanation is supported by the complete recovery of nerval function following wound heailing.

On the contrary, IONM signal was abnormal in the two cases with permanent RLNI. In the first case, the RLN was thermally damaged using a bipolar diathermy while trying to seal a bleeding vessel. The cause of permanent RLNI in the secound case could not found. These patients we referred to a logopedist.

Postoperative hemorrhage was seen in 3.2 % and in 3.4 % of the study and consultant group respectively. Postoperative bleeding was diagnosed based on the amount of blood in the drains. All cases of postoperative bleeding occurred within 12 h of surgery and were surgically managed. In two cases, both from the consultant group, the cause of hemorrhage was an open vascular stump. In both cases, the insufficiently sealed vessels were of medium size. Postoperative hemorrhage therefore could not be blamed on the size of the vessel per se. This is true for the device (Ligasure). The cause of postoperative bleeding in these cases therefore, must be at the level of application. This opinion is supported by the fact that postoperative hemorrhage has not been recorded in our department since the introduction of a double-sealing technique, i.e. double application of the LigaSure to produce two coagulation zones at the proximal end of the vessel before vascular division.

Although both groups were similar in terms of diagnosis and operative procedure, consultants managed a significant number of large goiters. This constitutes a selection bias, which is reasonable in a teaching center without jeopardizing surgical outcome.

The results presented in this study are based on the usage of a single sealing device, i.e. the LigaSure. It is thinkable, that similar effects could be achieved with other sealing devices like the harmonic scapel.

Taken together, although surgery lasted significantly longer when performed by trainees, there was no difference in outcome between patients managed by experienced consulting surgeons and those managed by surgical trainees. Therefore, sutureless thyroid surgery could be safely performed by surgical trainees without relevant increase in perioperative complications and thus could be safely integrated in endocrine surgical training programs.

The main limitations of this study include the small size of the study population and its retrospective nature. Besides, patient selection was not blinded with some degree of bais with regard patient allocation was inevitable to ensure patient safety. Therefore the trend reported in this study must be validated in studies with a better design and large population.

## Conclusions

While sutureless thyroid surgery was safely performed by surgical trainees without relevant increase in perioperative complications in our department, further larger scale studies would be needed to confirm this would be the case more widely.

## Abbreviations

RNL: recurrent laryngeal nerve
RLNI: recurrent laryngeal lerve injury
ASA: American Society of Anesthesiologists
IONM: intra operative neuro-monitoring
Min: minutes
Ml: milliliters
